# Refined Voting and Scene Feature Fusion for 3D Object Detection in Point Clouds

**DOI:** 10.1155/2022/3023934

**Published:** 2022-12-29

**Authors:** Hang Yu, Jinhe Su, Yingchao Piao, Guorong Cai, Yangbin Lin, Niansheng Liu, Weiquan Liu

**Affiliations:** ^1^The School of Computer Engineering, Jimei University, Xiamen 361021, China; ^2^Computer Network Information Center, Chinese Academy of Sciences, Beijing, China; ^3^Fujian Key Laboratory of Sensing and Computing for Smart Cities, School of Informatics, Xiamen University, Xiamen 361005, China

## Abstract

An essential task for 3D visual world understanding is 3D object detection in lidar point clouds. To predict directly bounding box parameters from point clouds, existing voting-based methods use Hough voting to obtain the centroid of each object. However, it may be difficult for the inaccurately voted centers to regress boxes accurately, leading to the generation of redundant bounding boxes. For objects in indoor scenes, there are several co-occurrence patterns for objects in indoor scenes. Concurrently, semantic relations between object layouts and scenes can be used as prior context to guide object detection. We propose a simple, yet effective network, RSFF-Net, which adds refined voting and scene feature fusion for indoor 3D object detection. The RSFF-Net consists of three modules: geometric function, refined voting, and scene constraint. First, a geometric function module is used to capture the geometric features of the nearest object of the voted points. Then, the coarse votes are revoted by a refined voting module, which is based on the fused feature between the coarse votes and geometric features. Finally, a scene constraint module is used to add the association information between candidate objects and scenes. RSFF-Net achieves competitive results on indoor 3D object detection benchmarks: ScanNet V2 and SUN RGB-D.

## 1. Introduction

3D object detection aims to locate and recognize 3D objects from point clouds, playing an important role in visual recognition. Compared with 2D images, point clouds could describe the precise geometric shapes of 3D objects and they are robust to various environments, such as illumination changes and background changes. Therefore, predicting 3D bounding boxes using the point clouds in real-world environments is of high practical value, which is used in fields such as modal detection [1], AI navigation [2], indoor robot navigation [3], 3D cadastre models [4], and robot grasping [5, 6].

Driven by advances in 2D images using convolutional neural networks (CNNs), several architectures, such as DSS [1] and 2D-driven method [7], have been proposed for object detection in point clouds. These methods, applying image-based feature extraction techniques, project point clouds onto multiperspective views. One of the major limitations of multiview-based methods is loss of height data and 3D shape information, which markedly decreases the accuracy of the bounding box regression branch. Other methods, such as 3D ConvNet [8], VoxelNet [9], and PV-RCNN [10], divide point clouds into equally distributed grids, which usually contain an uneven number of points due to the sparsity of point clouds. Inspired by the success of PointNet [11] and its variants in object classification and semantic segmentation, instead of converting irregular point clouds to grids, point-based approaches, such as VoteNet [12], MLCVNet [13], HGNet [14], and BRNet [15], process raw points directly to learn 3D representations.

VoteNet [12] is a detection framework that uses the Hough voting strategy [3]. First, VoteNet samples seed points by feeding a full point cloud into PointNet and then, applying a deep neural network, uses seed points to return the center of objects. Finally, the voted centers are then used to generate box proposals. However, two notable limitations to VoteNet still exist. (1) Voted centers are poorly located and lack geometric information. A voted center in VoteNet is a vital point, where a bounding box is located. By contrast, seed points generated by PointNet++ and distributed on the surface of an object have the geometric information of the objects. (2) The proposed module groups and predicts every object individually, without taking into account global scene features and local relationships between those objects. Compared with an outdoor scene, in an indoor scene there are several co-occurrence patterns for objects, such as bathtub, shower curtain, toilet in a bathroom; tables and chairs in a conference room; and bed and cabinet in a bedroom. We conclude that those scene co-occurrence patterns, which improve object detection performance, are critical for 3D understanding. A natural way to use these co-occurrence patterns is to design a module to fuse the local relations between objects and global scene features.

In summary, we propose a novel voting-based indoor 3D object detection method, refined voting and scene feature fusion network (RSFF-Net), which incorporates end-to-end learnable attention supervised feature enhancement into a voting-based framework. For each coarse vote, a fixed number of points from original point clouds is sampled randomly to create geometric object information. Then, a refined voting module combines the coarse votes with the features of resampled points to take a second vote to improve voted center quality. Inspired by the idea of scene context in 2D object detection, we add global scene context to model the semantic association between scenes and objects, such as a living room having a sofa, a study room having a bookcase, and a bathroom having a toilet.

**Figure 1:**
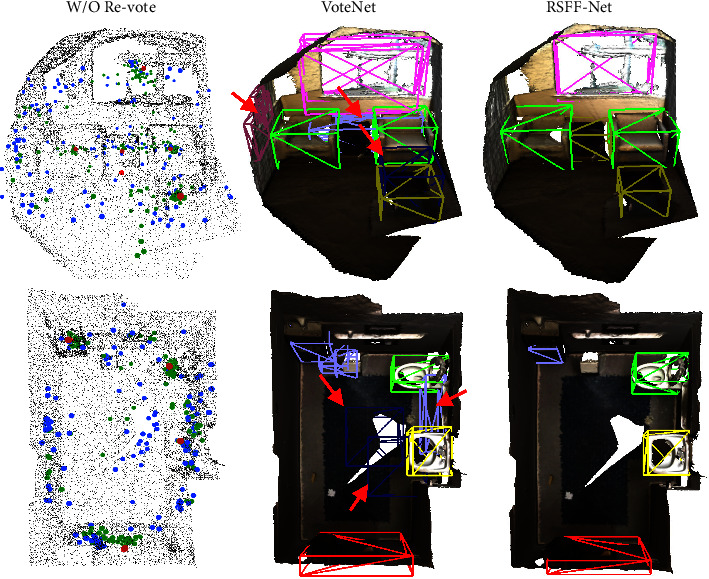
Illustrations of the effectiveness of refined voting.

The effectiveness of refined voting is illustrated in Figure 1. In the first column (W/O Revote), red points denote ground truth centers; blue and green points denote the vote results of VoteNet and our proposed RSFF-Net, respectively. Many blue points deviate from the center points, which are denser after refined voting (green points) near the ground truth than those of the single voting network. In the second column (VoteNet), red arrows mark false detections and false detection boxes. In the third column (RSFF-Net), bounding boxes of different colors represent different object categories. The predicted bounding boxes (pink and red boxes) of the proposed RSFF-Net fit better than those of the previous VoteNet due to the improved accuracy of the voted object centers. The number of certain duplicate boxes (purple boxes) was reduced, and the number of false detections (dark blue boxes) decreased.

The specific contributions of this paper are as follows:

- We propose a novel end-to-end 3D object detection framework, RSFF-Net, to address the central voting error and small object detection. The proposed RSFF-Net generates local voting attention regions, reliably reselects voting points from seed points, and then trains and refines object proposals to achieve more robust object classification and more accurate bounding boxes.
- We design three novel modules to fuse features from both seeds and votes. These modules ensure that the central point being voted obtains a comprehensive merged feature. These modules also use semantic relations between object layouts and scenes to refine proposals.
- To demonstrate the effectiveness of our proposed modules, extensive experiments were conducted with the ScanNet V2 [16] and SUN RGB-D [17] datasets. In addition, the proposed RSFF-Net performs better on small objects than VoteNet.

## 2. Related Work

### 2.1. 3D Object Detection

Recently, exciting breakthroughs have been made in 3D object detection using deep convolutional networks. Current mainstream detector frameworks can be roughly categorized into three primary types based on their preprocessing methods on original point clouds: voxel grid [9, 10, 18, 19], bird's eye view [20–24], and points [12–15, 25, 26].

#### 2.1.1. Voxel Grid

Voxel-based approaches convert irregular point clouds into 3D voxels [9, 18, 19, 27, 28]. VoxelNet [9] divides a point cloud into equally spaced 3D voxels, applies multilayer perceptrons (MLPs) to points, and obtains a unified feature representation in each voxel. In [27, 28], the authors encoded each non-empty voxel with six statistical quantities and fused multiple local statistics to represent each voxel. HVNet [19] and Voxel-FPN [18] aggregate a set of multiscale voxel features generated by voxelization from various voxel sizes. PV-RCNN [10] combines a keypoint feature with a voxel feature to obtain accurate location information. Supervoxels [29–31] use a novel supervoxel segmentation algorithm to enhance road boundaries from 3D point clouds. Voxel-based methods often use computationally inefficient 3D sparse convolutions to extract features from the voxel representation.

#### 2.1.2. Bird's Eye View

In contrast to building voxel grids, many existing studies render cloud points into 2D regular lattices [20, 21, 23], project the points onto bird's eye view (BEV) images, and extract features with 2D convolutional layers. MV3D [21] introduces a 3D object proposal generation module and a multiview encoding scheme to combine region-wise features. AVOD [32] also consists of two networks: region proposal and prediction. The region proposal network must perform multimodal feature fusion on high-resolution feature maps. In [20, 24], the authors projected a point cloud onto a 2D BEV image and a proposal-free single-stage detector. These handcrafted BEV methods easily achieve stable, efficient speed but sacrifice accuracy, which is limited by coarse-grained point cloud representations.

#### 2.1.3. Point-Based Method

Many methods [12–15, 25, 33–35] have used PointNet++ as the backbone network to directly extract features from unordered point cloud for 3D object detection. VoteNet [12] votes for group points to a center point based on learned seed features from PointNet++. This method and its variants [13, 14, 25] yield excellent results. MLCVNet [13] introduces multilevel contextual information into the voting stages to equip the network with the ability to learn object-level and global-level context. HGNet [14] uses a hierarchical graph network to capture the relationship between center points. By optimizing votes and feature fusion between points, FFRNet [35] improves object detection. 3DSSD [25] uses the FPS sampling strategy to decrease inference time. BRNet [15] backtraces the representative points and revisits seed points to better capture local structural features. To equip the network with the ability to learn object-level and global-level context, Pan et al. [33] designed two transformer modules to learn context-aware representations at the object and scene levels. H3DNet [34] defines a hybrid set of geometric primitives and refines the bounding boxes by an overcomplete set of constraints generated by those geometric primitives. However, the effects of the above networks are general in complex and changeable indoor scenes containing many details.

### 2.2. Attention-Based Network

Inspired by the idea of self-attention in natural language processing [36], recent studies have applied self-attention mechanisms to improve scene understanding by modeling the relationships between objects [13, 37–39]. For example, in [40], for 2D vision, an attention-based method was proposed for joint visual language modeling. Recently, DETR [41] employed a transformer for 2D object detection and achieved excellent performance. Regarding 3D point data processing, the work in [39] uses a point context attention network that encodes local features into global features to capture the contextual information in 3D points.

Conversely, PCAN [38] proposes a point attention transformer to process point clouds. When detecting 3D objects in large-scale point clouds, in [42], an attention-based PointNet is proposed to find regions of interest instead of processing the entire scene. MLCVNet [13] learns multilevel contextual information between patches, objects, and scenes. HGNet [14] uses multilevel semantic information and shape attention graph convolution to capture shape information from the original point clouds. VoTr [43] uses a self-attention mechanism to solve the limitation of the receptive field size of voxel grids and establishes long-distance perceptual relationships between voxels. Based on multiple ranges of attention networks, Pointformer [33] designs a novel backbone network for 3D point clouds. Attentional-PointNet [42] uses an attention mechanism to classify each small area in the three-dimensional space. 3DETR [44] applies attention operations in disordered point clouds to capture remote context information. Previous methods mainly used attention networks to learn the relationships between points or find concerned local regions. Our aim is to use an attention mechanism to capture the semantic information between a global scene and its objects.

## 3. Materials and Methods

### 3.1. Overview

**Figure 2:**
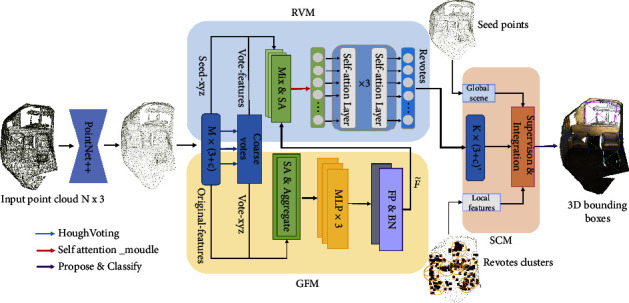
The overall architecture of the proposed RSFF-Net.

Geometric function module (GFM), refined voting module (RVM), and scene constraint module (SCM) are proposed to encode the geometric features, revote the object center, and optimize proposals with local and global semantic association constraints. The overall architecture of the proposed RSFF-Net is shown in Figure 2.

In voting-based methods, subsampling strategies may corrupt the spatial geometry of an object in a point cloud. Prior learned seed features severely affect the accuracy of voted centers. The vote clustering operation for an individual object also ignores the relationships between objects. Therefore, to improve the accuracy of voting centers and integrate local-global association information into the proposal network, we propose a novel voting-based method, RSFF-Net, for refined voting and scene feature fusion operations. Taking an unordered set of 3D points as input, the proposed RSFF-Net outputs a set of object bounding boxes, *B*; each box, *b*∈*B*, is associated with a predefined category label, a center location, the size of the bounding boxes, and the orientation. As shown in Figure 2, RSFF-Net consists of three primary modules: GFM, RVM, and SCM.

To extract point features from irregular point clouds, we use PointNet++ to generate seed points. Next, a Hough voting operation predicts coarse voting points. Then, GFM resamples original points near coarse votes and learns object structural features for refined voting. RVM takes a second Hough voting after combining coarse voting and the feature of revisit points to output refined virtual center points. The RVM module helps to accurately locate a 3D bounding box and reduce overlapping bounding boxes. SCM uses an attention network to integrate global scene context with auxiliary proposal clustering.

### 3.2. Coarse Voting Module

In a 2D image, an object center must be a real pixel having a rich texture. However, in 3D point clouds, the object center is typically far from the surface of the object and cannot be scanned by a data collection device. Thus, we generate new virtual points to represent object centers using an evolved version of 3D voting, which is inspired by the Hough voting framework [39].

We use PointNet++ to learn multidimensional features from initial point clouds *P*_input_. The backbone generates the seed points *S* = {*s*_*i*_}_*i*=1_^*M*^, where *s*_*i*_ is the *i*-th point in seed points. Every seed has features [*x*_*i*_, *f*_*i*_]∈*R*^(3+*c*)^, where 3+*C* represents the three-dimensional coordinates and feature information from its surrounding points within a radius, respectively. Specifically, the structure of PointNet++ consists of several set abstract (SA) layers and feature propagation layers, in which the parameters refer to the point cloud feature learning backbone network in VoteNet. The voting block uses the point patches with seed features as input and regresses the coarse votes *V*_1_ = {*r*_*i*_}_*i*=1_^*M*^, where *r*_*i*_, the *i*-th point in vote points, has features [*x*_*i*_, *f*_*i*_]∈*R*^(3+*c*)^. Coarse vote prediction is performed by a multilayer perceptron.

### 3.3. Geometric Function Module

In VoteNet, 1,024 seed points represent the characteristics of the whole set of the input point clouds. The coarse votes are derived from the seeds, which ignore the details of a single object. Hence, to enhance the object features and learn the potential geometric features of a single object, we resample some original points around the coarse votes.

We first use farthest point sampling to sample uniform reference points *R*={*r*_*i*_}_*i*=1_^*p*^ based on coarse votes. Returning to the original points, we use a minor modified K-nearest point sampling strategy, which adds the distance from object center to select the revisited points around reference points. We obtain the local point set, *P*_*i*_={*p*_1_, *p*_2_,…, *p*_*k*_}, from the original points near the reference point, *r*_*i*_. We label point, *p*_*i*_, in the revisited set *P* as follows: the distance between the point and the nearest object center is less than 0.3, and the original point is also the *k*-th point closest to the reference point, *r*_*i*_. After that, with the ReLU activation function, the network learns the geometric features of an object from revisited points using three MLPs. The module takes the coordinates of reference points *r*_*i*_ and the features of revisited point sets *P*_*i*_ as input and outputs the learned features $\tilde{f_{i}}$ of *r*_*i*_.

### 3.4. Refined Voting Module

We place the fused features, $\tilde{f_{i}}$, the revisited points, *P*_*i*_, and the coarse votes, *R*, into the MLP for feature fusion. Similar to the Hough voting framework, RVM replaces set abstraction layers with self-attention feature propagation. We adjusted the number of convolutional channels and embedding method of the original voting layer, which uses an attention mechanism to learn the local information of a point. With Euclidean space offset, ∆*x*_*i*_ ∈ *R*^3^, and feature offset, ∆*f*_*i*_ ∈ *R*^*c*^, MLP returns revotes, *v*_*i*_=[*x*_*i*_, *f*_*i*_], from the mixed points, $x_{i}=\tilde{x_{i}}+∆x_{i}$, $f_{i}=\tilde{f_{i}}+∆f_{i}$. The predicted 3D offset, ∆*x*_*i*_, is supervised explicitly by the following regression loss:

(1) $$\begin{array}{c} L_{Rvote-reg}=\frac{1}{M_{pos}}\underset{i}{\sum }\left\|∆x_{i}-∆x_{i}^{*}\right\|\;1\left[s_{i}on\;object\right]\text{,}\\ ∆x_{i}=\alpha ∆vx_{i}+\beta ∆sx_{i}\text{,} \end{array}$$

where 1[*s*_*i*_ on object] indicates whether a seed point is on the surface of an object; *M*_*pos*_ is the count of the total number of revoting points on the surface of an object surface; ∆*x*_*i*_^*∗*^ is the ground truth displacement from the revote position *x*_*i*_ to the bounding box center of the object to which it belongs; ∆*vx*_*i*_ is the offest of coarse votes; ∆*sx*_*i*_ is the offset of seeds; and *α* and *β* are hyperparameters.

### 3.5. Scene Constraint Module

In an indoor scene, there are several common senses for object layout, such as bookcases appearing in a study room, sofas in a living room, and toilets in a bathroom. The indoor objects also have strong mutual semantic associations, which can be used as a priori information for indoor object detection. Inspired by the idea of scene context extraction in [45], we propose a scene constraint module (SCM), which uses global scene context information to improve the performance of the bounding box proposal and classification.

**Figure 3:**
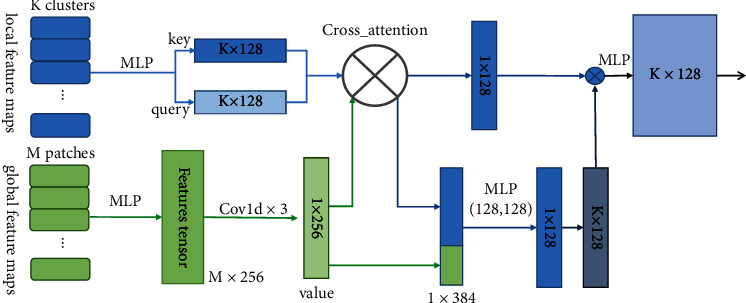
Details of the architecture of the scene constraint module.

Figure 3 shows the detailed composition of SCM. An attention coding design modified for the global scene is used to learn the semantic association between objects and scenes. SCM uses global features from original points and object-level local feature clusters from revoting to create a new branch; then, the module applies a cross-attention mechanism to model objects and scenes.

Given a set of revotes, {*v*_*i*_ = [*x*_*i*_; *f*_*i*_] ∈ *R*^3+*c*^}_*i*=1_^*M*^, we sample *K* revotes as the refined vote centers by farthest point sampling. Then, we generate *K* clusters by grouping *K*-nearest neighbors of each cluster center and learn cluster features by several MLPs. Each cluster, *C*_*i*_ = [*X*_*i*_, *F*_*i*_], is sent to the MLPs, and then, 1 × 1 convolution is used to form a single vector representing the cluster as the key and query. We introduced global feature patches, *P* = {*p*_1_, *p*_2_,…, *p*_*M*_}, from seeds to obtain a vector after convolution and max-pooling and then fed the vector into the self-attention module with the key and query values to generate a new feature map. The encoding of supervision relationships is summarized as follows:

(2) $$\begin{array}{c} C_{super_{i}}=MLP\left(\left[CP_{i};MLP\left(C_{i}\right)+\max_{i=1,\ldots ,n}\left\{MLP\left(P_{i}\right)\right\}\right]\right)\text{,}\\ CP_{i}=Attention\left(\left[MLP\left(C_{i}\right);\max_{i=1,\ldots ,n}\left\{MLP\left(P_{i}\right)\right\}\right]\right)\text{,} \end{array}$$

where*i* = 1,…, *k*, *C*_super_*i*__ is the *i*th cluster, and Attention (∙) is the attention mapping of CGNL [46].

### 3.6. Proposal and Classification

After grouping, we use a network to generate bounding boxes and classification. Given *C*_super_*i*__ with *Z*_*i*_ ∈ *R*^3^ as the center location and *H*_*i*_ ∈ *R*^*D*^ as the cluster feature, an object proposal for this cluster *p*(*C*) is generated by passing the set input through a PointNet-like module as follows:

(3) $$\begin{array}{c} p\left(C\right)=MLP_{2}\left\{\max_{i=1,\ldots ,n}\;\left\{MLP_{1}\left(\left[Z_{i}^{\prime };H_{i}\right]\right)\right\}\right\} \end{array}$$

(4) $$\begin{array}{c} \frac{Z_{i}^{\prime }=\left(Z_{i}-Z_{j}\right)}{r}. \end{array}$$

In equation (3), the feature point set from each candidate is processed independently by *MLP*_1_. Then, decoding information for detection and classification is extracted, maximally pooled (by channel) to a single vector, and passed to *MLP*_2_ for prediction. The information of refined voting points is further combined and scored. To obtain the standard coordinate proposal, we convert the voting position, by equation (4), into a local standardized coordinate system. The proposal *p(C)* contains five parameters (center, heading, scale, objectness, and category) to describe the bounding box.

We use cross-entropy loss to supervise the objectivity scores of negative proposals near the center (within 0.3 m) of the ground truth object or far from (above 0.6 m) any center. For positive proposals, we further supervise the bounding box estimation and class prediction bounding box based on ground truth. Specifically, we follow the method described in VoteNet, which decouples box loss to center regression, heading angle estimation, and box size estimation. For semantic classification, we also use cross-entropy loss. In the regression of all detection losses, we use Huber (smooth-L1 [47]) loss.

## 4. Results and Discussion

In this section, we first describe the dataset used in our experiments and the experimental setup. Then, several ablation studies were conducted to demonstrate the superiority of the proposed module in RSFF-Net. Finally, the compared baselines and experimental results on ScanNet V2 [16] and SUN RGB-D [17] datasets are used to demonstrate the superiority of the proposed RSSF-Net.

### 4.1. Datasets

#### 4.1.1. ScanNet V2 Dataset

ScanNet V2 [16] is a richly annotated dataset of 3D reconstructed meshes of indoor scenes, which contains about 1,200 training examples collected from hundreds of different rooms and is annotated with semantic and instance segmentation for eighteen object categories. We sampled vertices from the reconstructed meshes as the input in point clouds. Because ScanNet V2 does not provide oriented bounding box annotation, as in [21], we predict axis-aligned bounding boxes instead. The inputs for the proposed RSSF-Net are randomly subsampled points from the raw data (i.e., 40,000 points from a 3D mesh in the ScanNet V2 dataset).

#### 4.1.2. SUN RGB-D Dataset

SUN RGB-D [17] is a single-view RGB-D dataset used for research on 3D scene understanding that contains 10,335 indoor RGB and corresponding depth images. The RGB images are aligned with the depth channel and used to query the corresponding image area from the 3D point scene. Each point in the point cloud has a semantic label and an object bounding box. There are 37 types of annotated objects in the dataset. We trained and recorded the results from the ten most common categories, which are the same as those for VoteNet.

### 4.2. Experimental Setup

Inputs of RSSF-Net are the randomly downsampled point clouds, containing 20 k points for the SUN RGB-D dataset and 40 k for the ScanNet V2 dataset. In addition to XYZ coordinates, each point contains a height feature, indicating its distance to the ground. Floor height is estimated to be 1% of the height of all points. To increase the training data, we randomly subsampled data from a field point cloud. Point cloud data are randomly flipped in two horizontal directions, randomly rotated on the vertical axis by [−5°, 5°], and scaled randomly by [0.9, 1.1]. The end-to-end model, RSSF-Net, is trained by using the Adam optimizer with a batch size of 8. The base learning rate was 0.005 for the ScanNet V2 dataset. RSSF-Net was trained for 180 epochs on both datasets. To verify timeliness, we referred to the comparison method based on the PyTorch platform equipped with two NVIDIA GeForce RTX 2080 Ti GPUs, which require approximately 4.5 hours to train the model with the ScanNet V2 dataset until convergence and approximately eleven hours with the SUN RGB-D dataset.

### 4.3. Ablation Study

#### 4.3.1. Individual and Combined Effects of Submodules

To quantitatively evaluate the effectiveness of the proposed contextual submodules of RSSF-Net, we performed experiments with different combinations of these modules. The baseline method was VoteNet. Then, we added the proposed submodules one by one into the baseline model. Applying the GFM, RVM, and SCM modules led to the following improvements in mAP@0.25: 3, 4.1, and 3.8, respectively. The results of the different combinations of the three modules are detailed in Table 1, with the highest mAP@0.25 score being 65.9.

We tested the combined work effectiveness of the three modules and found that, with the cooperation of the RVM module, the performance of the network improved significantly. Supplementing the geometric information of the proposal in the original point cloud helps improve the revoted center point by 1.8 points. SCM helps to judge the semantic category of the proposal by infiltrating the scene layout information into the proposal's learning of the surrounding environment and, simultaneously, improves the quality of the proposal's center point.

#### 4.3.2. The Effect of Submodule Location

**Figure 4:**
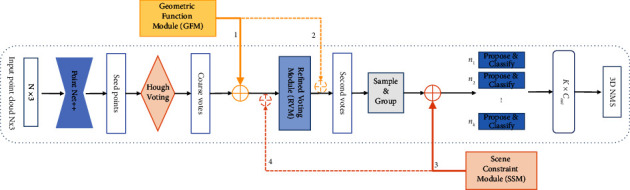
Locations where the proposed geometric function module (GFM), refined voting module (RVM), and scene constraint module (SCM) can be inserted.

In addition, we also perform a detailed ablation study to analyze the effect of the proposed three submodules when placed in different positions. As shown in Figure 4, the solid arrows indicate the best combinations of positions for the proposed method, and the dashed lines indicate the adjusted positions of the GFM and SCM. Yellow and orange solid arrows (1, 3) indicate the positions of GFM and SCM in the RSFF-Net; dashed arrows (2, 4) indicate alternative positions for GFN and SCM.

The quantitative results are shown in Table 2. We considered placing SCM after the coarse voting or refined voting and determined its performance separately. From the results, SCM is more effective after refined voting. Global feature supervision of the candidates effectively manages the features between candidates and supervises the detection and classification of the candidates, eliminating results with large deviations, while considering the rules that must be followed for objects in indoor scenes. The global scene also assists in the classification of objects (i.e., there is typically a bed in a bedroom; a bathtub typically appears only in a bathroom).

### 4.4. Result Comparison

We compare the proposed RSFF-Net with several existing methods, including the following: F-PointNet [48], GSPN [49], VoteNet [12], 3DSIS [50], HGNet [14], MLCVNet [13], H3DNet [34], RandomRooms [51], 3DETR [44], BRNet [15], Pointformer [33], and DisARM [52], with the ScanNet V2 validation set. Detailed results are shown in Table 3. The mAP@0.25 of the proposed RSFF-Net reached 65.9%, which is 7.3% higher than that of VoteNet; mAP@0.50 was even higher, showing an increase of 11.4%.

There are differences when H3DNet processed the SUN RGB-D dataset (e.g., H3DNet subsampled 40,000 points from each scene for input, while other methods used 20,000 points). In addition, H3DNet reported only the results of each category when using PointNet++ as the backbone network. Other comparison methods used only PointNet++ as the backbone network. The proposed RSFF-Net also achieves a marginal improvement of 1.5 in terms of mAP@0.25 over H3DNet, even when using a different backbone.

We also compared our methods with several baseline methods on the SUN RGB-D dataset. The results are given in Table 4, which shows that the proposed RSFF-Net achieves performance comparable with most existing methods. When considering a point cloud only, VoteNet obtained a detection accuracy of 57.7 in terms of mAP@0.25. Note that the proposed RSFF-Net provides a marked absolute gain of 3.6 compared to VoteNet. Despite the differences in the datasets, RSFF-Net still outperforms, improving by 3.6% and 10.7% on mAP@0.25 and mAP@0.5, respectively. We observe that on the two datasets, in the case of mAP@0.5, the performance of RSFF-Net is superior, indicating that when the IoU is 0.5, our method provides more high-quality proposals than VoteNet. The location is more accurate and efficient, as fully reflected by our experimental results.

In Tables 5 and 6, we show the respective dataset accuracies under the semantic categories.  Table 5 shows the detailed performance scores under the semantic categories for ScanNet V2 (i.e., for each object category in the ScanNet V2 dataset, mAP@0.25). For certain specific categories, such as bookshelves, doors, and windows, which are similar to flat objects, generally large improvements were obtained, increasing by 15.4, 12.3, and 10 points compared to VoteNet. The reason for these results may be that these objects have regular geometric edges, which can be better supplemented, making the features learned by refined voting more accurate. In addition, these objects have special semantic classes and unique locations. The SCM is more sensitive to the layout information of these objects in the global scene, so it is more sensitive in detection.

When presenting the 3D object detection results for the SUN RGB-D validation dataset, we assessed performance using the SUN RGB-D V1 data to make a fair comparison with existing methods. As shown in Table 6, the proposed RSFF-Net achieved the best performance on mAP@0.25 on 7 out of 10 of the classes from the SUN RGB-D dataset. The proposed RSFF-Net also had a visible effect on bathtub, desk, and bookshelf, which increased by 9.4, 5.6, and 7.6 points, respectively. These objects all have a strong relationship with the scene, indicating that the semantics of the upper and lower levels are instructive and helpful to the detection.

### 4.5. Results with ScanNet Dataset

**Figure 5:**
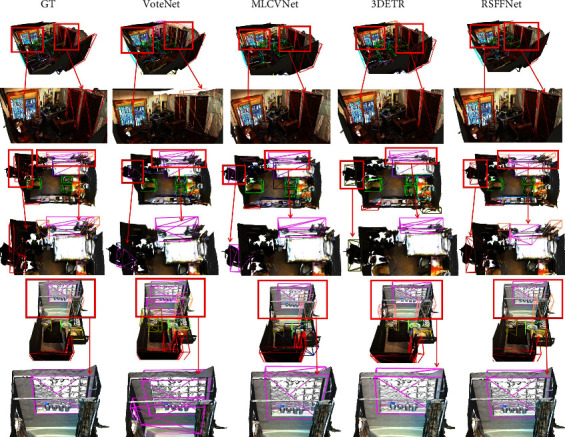
Comparison of object detection results between VoteNet [12], MLCVNet [13], 3DETR [44], and the proposed RSFF-Net for objects embedded in the wall, which are similar to the wall. Bounding boxes of different colors represent different object categories.

Many objects, such as windows, doors, and pictures, which are embedded in or attached to walls in indoor scenes, are typically markedly different from walls in RGB images. However, these objects appear on the surface of walls and are easily confused with walls in pure point clouds. Thus, the objects are easily incorrectly detected by detectors without RGB image inputs. As shown in Figure 5, all doors and windows in three images are embedded in the walls. VoteNet exhibits relatively poor performance in all three scenes.

As shown in the first scene in Figure 5, both doors and windows are classified inaccurately, and several invalid boxes are predicted in the top right corner. In the second scene, VoteNet [12] also classifies a door as a window and misses the curtains near the window. Also, duplicate boxes are generated for the window and door in the third scene. Although detecting windows and doors correctly in the first two scenes, MLCVNet [13] also classifies the door incorrectly in the second scene and creates one additional box for the door in the third scene. Although detecting correctly in the first and third scenes, 3DETR [44] incorrectly detects the door as a locker in the second scene. In contrast, the proposed RSFF-Net successfully recognizes the doors and windows in all three scenes and also correctly detects the window curtains in the second scene, whereas both VoteNet and MLCVNet tend to miss the curtains.

Additionally, in contrast to the other two methods, according to the partially enlarged image, the boxes predicted by the proposed RSFF-Net fit much better around the real objects. For example, while MLCVNet, 3DETR, and RSFF-Net all detect the glass doors in the first scenario of Figure 5, it is clear that the bounding box of RSFF-Net is more perfect. A possible reason for the proposed RSFF-Net being able to effectively reduce duplicate and empty bounding boxes is that the proposed refined voting module improves center point location and directs the network to pay attention to the correct regions. The proposed RSFF-Net also moves adjacent points to the centers, which helps remove duplicate boxes during the non-maximum suppression (NMS) operation. In some narrow rooms, such as living room and bathroom, each scene has several object categories with a large difference in object size and geometry.

**Figure 6:**
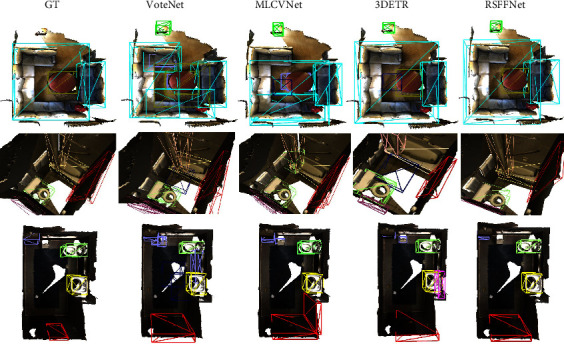
Comparison of object detection results between VoteNet [12], MLCVNet [13], and 3DETR [44]. Additionally, the proposed RSFF-Net achieves acceptable results for objects of various sizes and shapes in a special room. Bounding boxes of different colors represent different object categories.

Different objects are distributed in a specific form and have strong semantic relations with each other. The objects, such as sofas and coffee tables, and toilets and bathtubs frequently appear in pairs.  Figure 6 shows that RSFF-Net exhibits a strong ability to improve detection precision in those scenarios. In the first two scenes, the proposed RSFF-Net outputs only a total of nine bounding boxes for eight objects, whereas VoteNet, MLCVNet, and 3DETR output eighteen, fifteen, and twelve bounding boxes, respectively. Accuracy of the boxes is also better with the proposed RSFF-Net. Again, it is reasonable to assume that this improvement is related to the benefits of the refined voting module. Considering bounding box quality, the proposed RSFF-Net achieves a nearly perfect result for the sofa (light blue boxes), bathtub (yellow box), toilet (light green), and door (red box). In contrast, by producing low-quality boxes, both VoteNet and MLCNet generate an inadequate box for the larger sofa in the first scene and, in the second scene, produce duplicate boxes for the door; VoteNet, MLCVNet, and 3DETR produce several invalid boxes (dark blue) in empty areas. Both MLCVNet and the proposed RSFF-Net achieve acceptable results for the toilet, bathtub, and shower curtain.

**Figure 7:**
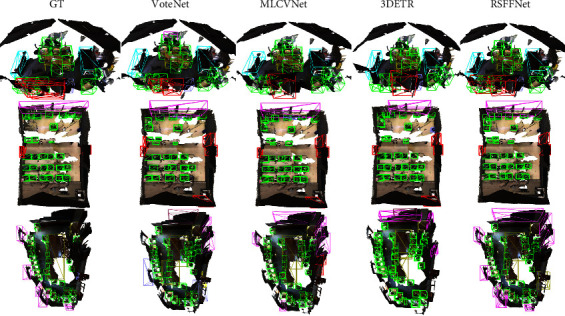
Comparison of object detection results between VoteNet [12], MLCVNet [13], 3DETR [44], and the proposed RSFF-Net for densely distributed objects. Bounding boxes of different colors represent different object categories.

Sometimes, indoor scenes contain densely packed objects in certain regions. During inference, plenty of centers in an image are of the same category, thereby increasing the difficulty of individual detection. Detailed results are shown in Figure 7. In all three scenes, both 3DETR and RSFF-Net separate the rows of chairs. However, VoteNet misses several chairs in the center region of the second scene and misses a few windows in the third scene. Overall, in this situation, both VoteNet and MLCNet are prone to generating redundant boxes in this situation. Also, all three methods predict two chairs in the top left of the second scene, even though they are not labeled in the ground truth.

**Figure 8:**
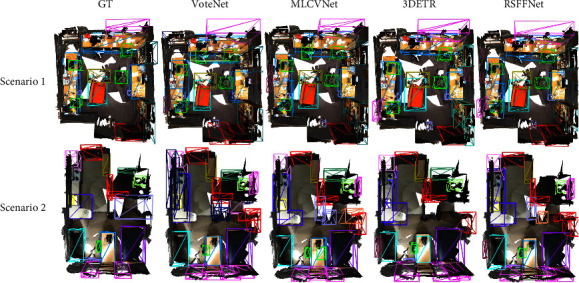
Comparison of object detection results between VoteNet [12], MLCVNet [13], 3DETR [44], and the proposed RSFF-Net for multifunctional scenes. Bounding boxes of different colors represent different object categories.

In the multifunctional scenes, the proposed RSFF-Net does not show a better result than other detectors (see Figure 8). From a functional point of view, a room can be regarded as a study room or a living room. In Scenario 1, there are three tables, one sofa, several chairs, and many objects on tables. Cluttered objects that cover the tables become noise data and make it even difficult to extract key feature for tables. Therefore, for all three methods, the results for the tables are poor. Similarly, Scenario 2 can be seen as the combination of kitchen and living room. Only two windows are embedded in the bottom of the scene. Owing to the bookshelf-like object between the windows, all methods generated redundant boxes for them. None of the three detectors correctly detected the furniture in the right center of the room (marked in light blue). Two possible reasons for this are the lack of training samples and the occlusion of part of the object.

### 4.6. Results with SUN RGB-D

**Figure 9:**
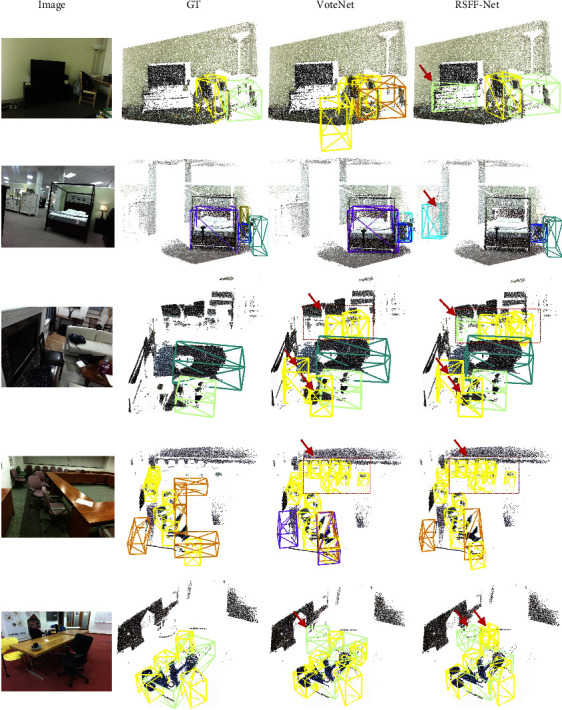
Qualitative results of the SUN RGB-D dataset. Boxes of different colors represent different types of objects. The bounding boxes pointed by red arrows denote the correctly detected objects that exist in RGB image but are unlabeled in point cloud. Bounding boxes of different colors represent different object categories.

Some qualitative results on the SUN RGB-D dataset are shown in Figure 9. Boxes of different colors represent different types of objects. The bounding boxes pointed by red arrows denote the correctly detected objects that exist in the RGB image but are unlabeled in the point cloud. Bounding boxes of different colors represent different object categories. As seen in Figure 9, the proposed RSFF-Net achieves promising results in a wide range of scenes including the bedroom, living room, and conference room. It is also noteworthy that almost all the objects in ground truth (GT) are detected correctly by our proposed RSFF-Net in those images, whereas VoteNet still has a few kinds of missed and false-positive detection. Additionally, many objects in the RGB image are not labeled or missing in the GT. For instance, the TV cabinet in the first scene is unlabeled and the chairs in last two scenes are only partially observed by the sensor. For those objects, RSFF-Net exhibits significant improvement over VoteNet, thereby demonstrating the effectiveness of the proposed approach.

## 5. Conclusion

3D object detection in indoor scenes is used in various AI environments. The proposed RSFF-Net introduces three novel modules to achieve better feature learning, center voting, and bounding box regression. The geometric function module attempts to add detailed object information for small objects caused by downsampling. Refined voting improves the accuracy of center points. Scene constraints introduce the relationships between a scene and its objects to improve classification accuracy. Compared with the several existing methods, the proposed RSFF-Net achieves a higher accuracy on both the ScanNet and SUN RGB-D datasets. In future work, we plan to apply these modules to other 3D scene understanding tasks, such as instance segmentation and 3D object reconstruction.

## Figures and Tables

**Table 1:** Ablation study on the ScanNet test dataset (the baseline work is VoteNet [12]).

Method	GFM	RVM	SCM	mAP@0.25	mAP@0.5
Baseline				58.6	33.5
—	✓			61.6	36.6
—		✓		62.7	40.6
—			✓	62.4	38.3
—	✓	✓		63.9	41.2
—	✓		✓	64.2	40.9
—		✓	✓	62.4	41.1
RSFF-Net	✓	✓	✓	**65.9**	**44.9**

**Table 2:** Ablation studies on the position of the proposed modules in the network (the proposed RSFF-Net is the baseline network).

Method	After voting	After revoting	mAP@0.25	mAP@0.5
Baseline			65.9	4 4.9
GFM		✓	63.6	42.4
SCM	✓		63.8	43.7

**Table 3:** 3D object detection results on the ScanNet V2 [16] validation set.

ScanNet V2	Input	mAP@0.25	mAP@0.5
DSS [1]	Geo + RGB	15.2	6.8
F-PointNet [48]	Geo + RGB	19.8	10.8
GSPN [49]	Geo + RGB	30.6	17.7
3DSIS [50]	Geo + 5 views	40.2	22.5
VoteNet [12]	Geo only	58.6	33.5
HGNet [14]	Geo only	61.3	34.4
MLCVNet [13]	Geo only	64.7	42.1
H3DNet (1BB) [34]	Geo only	64.4	43.4
Pointformer [33]	Geo only	64.1	—
3DETR [44]	Geo only	65.0	47.0
BRNet [15]	Geo only	**66.1**	**50.9**
DisARM [52]	Geo only	64.2	46.5
**RSFF-Net (ours)**	Geo only	65.9	44.9

Bold denotes the overall best result.

**Table 4:** 3D object detection results on the SUN RGB-D [17] validation set.

SUN RGB-D	Input	mAP@0.25	mAP@0.5
DSS [1]	Geo + RGB	42.1	—
COG [53]	Geo + RGB	47.6	—
2D-driven [7]	Geo + RGB	45.1	—
F-PointNet [48]	Geo + 5 views	54.0	—
VoteNet [12]	Geo only	57.7	32.9
HGNet [14]	Geo only	61.6	—
MLCVNet [13]	Geo only	59.8	—
H3DNet (4BB) [34]	Geo only	60.1	39.0
3DETR [44]	Geo only	59.1	32.7
Pointformer [33]	Geo only	61.1	—
BRNet [15]	Geo only	61.1	**43.7**
DisARM [52]	Geo only	**61.5**	—
**RSFF-Net (ours)**	Geo only	61.3	43.6

Bold denotes the overall best result.

**Table 5:** 3D object detection results on the ScanNet V2 validation set.

Methods	Cab	Bed	Chair	Sofa	Table	Door	Wind	Bkshf	Pic	Cntr	Fridg	Showr	Toiol	Sink	Bath	Ofurn	mAP@0.25
3DSIS [50]	19.8	69.7	66.2	71.8	36.1	30.6	10.9	27.3	0.0	10.0	53.8	36.0	87.6	43.0	84.3	16.2	40.2
3DSIS geo [50]	12.8	63.1	66.0	46.33	26.9	7.9	2.8	2.3	0.0	6.9	10.4	12.1	74.5	22.9	58.7	7.0	25.4
VoteNet [12]	36.3	87.9	88.7	89.6	58.8	47.3	38.1	44.6	7.8	56.1	45.4	57.1	94.9	54.7	92.1	37.2	58.7
RRVoteNet [51]	37.2	87.4	88.9	**89.8**	61.9	45.3	42.6	53.5	7.8	51.7	**67.2**	66.4	96.8	**62.6**	92.0	43.6	61.3
MLCVNet [13]	44.6	**89.6**	91.4	87.2	67.1	56.8	45.9	59.5	15.1	56.7	54.7	73.1	**97.8**	55.6	91.3	50.9	64.7
BRNet [15]	49.3	88.3	**91.9**	86.9	69.3	59.2	45.9	52.1	**15.3**	**72**	60.4	73.6	93.8	58.8	**92.2**	47.1	**66.1**
**Ours**	**50.7**	89.4	91.1	84.2	**70**	**58.9**	**48.1**	**60**	13.2	60.4	56.4	**81.3**	92.1	58.8	90.9	**51.6**	**65.9**

Bold denotes the overall best result.

**Table 6:** 3D object detection results on the SUN RGB-D validation set.

Methods	RGB	Bathtub	Bed	Bookshelf	Chair	Desk	Dresser	Nightstand	Sofa	Table	Toilet	mAP@0.25
DSS [1]	✓	44.2	78.8	11.9	61.2	20.5	6.4	15.4	53.5	50.3	78.9	42.1
COG [53]	✓	58.3	63.7	31.8	62.2	45.2	15.5	27.4	51.0	51.3	70.1	47.6
2D-driven [7]	✓	43.5	64.5	31.4	48.3	27.9	25.9	41.9	50.4	37.0	80.4	45.1
F-PointNet [48]	✓	43.3	81.1	33.3	64.2	24.7	32.0	58.1	61.1	51.1	**90.9**	54.0
VoteNet [12]	✓	70.0	82.8	27.6	73.1	23.2	27.2	60.7	63.7	48.0	86.9	56.3
MLCVNet [13]	×	79.2	85.8	31.9	**75.8**	26.5	33.3	61.5	**66.3**	50.4	89.1	59.8
**Ours**	×	**79.4**	**86.1**	**35.2**	74.9	**28.8**	**32.7**	**62.6**	66.1	**51.6**	90.4	**61.3**

Bold denotes the overall best result.

## Data Availability

The SUN RGB-D data used to support the findings of this study have been deposited in the SUN RGB-D repository (https://rgbd.cs.princeton.edu/). The ScanNet data used to support the findings of this study have been deposited in the ScanNet repository (https://www.scan-net.org/).
